# Prophylactic antibiotics for the prevention of cellulitis (erysipelas) of the leg: results of the U.K. Dermatology Clinical Trials Network’s PATCH II trial

**DOI:** 10.1111/j.1365-2133.2011.10586.x

**Published:** 2012-01

**Authors:** U.K Dermatology Clinical Trials Network's PATCH Trial Team

**Affiliations:** Centre of Evidence Based Dermatology, University of Nottingham, King’s Meadow CampusLenton Lane, Nottingham NG7 2N, U.K.

## Abstract

**Background:**

Cellulitis (erysipelas) of the leg is a common, painful infection of the skin and underlying tissue. Repeat episodes are frequent, cause significant morbidity and result in high health service costs.

**Objectives:**

To assess whether prophylactic antibiotics prescribed after an episode of cellulitis of the leg can prevent further episodes.

**Methods:**

Double-blind, randomized controlled trial including patients recently treated for an episode of leg cellulitis. Recruitment took place in 20 hospitals. Randomization was by computer-generated code, and treatments allocated by post from a central pharmacy. Participants were enrolled for a maximum of 3 years and received their randomized treatment for the first 6 months of this period.

**Results:**

Participants (*n* = 123) were randomized (31% of target due to slow recruitment). The majority (79%) had suffered one episode of cellulitis on entry into the study. The primary outcome of time to recurrence of cellulitis included all randomized participants and was blinded to treatment allocation. The hazard ratio (HR) showed that treatment with penicillin reduced the risk of recurrence by 47% [HR 0·53, 95% confidence interval (CI) 0·26–1·07, *P* = 0·08]. In the penicillin V group 12/60 (20%) had a repeat episode compared with 21/63 (33%) in the placebo group. This equates to a number needed to treat (NNT) of eight participants in order to prevent one repeat episode of cellulitis [95% CI NNT(harm) 48 to ∞ to NNT(benefit) 3]. We found no difference between the two groups in the number of participants with oedema, ulceration or related adverse events.

**Conclusions:**

Although this trial was limited by slow recruitment, and the result failed to achieve statistical significance, it provides the best evidence available to date for the prevention of recurrence of this debilitating condition.

Cellulitis (also known as erysipelas) of the leg is an acute, painful and potentially serious infection of the skin and subcutaneous tissue associated with significant morbidity^1,2^ and health costs (cost of inpatient admission alone was £96 million in 2009/2010).^3–5^ It is usually due to streptococcal infection.^6^ Risk factors for cellulitis of the leg include previous episode(s) of cellulitis; lymphoedema; toe web maceration; obesity and diabetes.^7–9^ Many patients have recurrent episodes (30–50%),^10,11^ which can lead to subsequent lymphoedema and ulceration.

Evidence for the use of prophylactic antibiotics to prevent further episodes is very limited. Three small randomized controlled trials (RCTs) hint at possible benefit (with 16, 40 and 58 participants, respectively).^12–14^ Some clinical guidelines recommend long-term antibiotic prophylaxis for patients with predisposing conditions.^15,16^ However, such recommendations are based largely on empirical evidence and clinical opinion is mixed.^17^

Two trials of cellulitis prophylaxis have been initiated by the U.K. Dermatology Clinical Trials Network. PATCH I has recruited to target and is due to report in 2012, and will assess the impact of 12 months of prophylactic phenoxymethylpenicillin (penicillin V) in patients with recurrent cellulitis (at least two previous episodes). The PATCH II trial, reported here, assesses whether 6 months of prophylaxis with penicillin V is effective in reducing repeat episodes of cellulitis of the leg in patients who have had one or more episodes.

## Methods

The PATCH II trial was approved by the Nottingham Research Ethics Committee 2 (reference: 06/Q2404/22); all participants gave written informed consent. Details of the trial were prospectively lodged in a trials register (ISRCTN03813200). A copy of the protocol and statistical analysis plan are available from the PATCH trial website (http://www.patchtrial.co.uk). A summary of the protocol has also been published.^18^

### Trial design

A double-blind, parallel group, RCT compared 6 months of penicillin V with placebo, in patients who had received treatment for cellulitis of the leg (both first episode and recurrent cases). As this was a prevention study, normal care was considered to be no treatment and so a placebo control group was an appropriate comparator. Participants were allocated on a 1 : 1 basis, and were followed up by telephone from the coordinating centre for a maximum of 36 months, depending on when they were enrolled.

Changes to the trial design after registration of the protocol, but prior to start of recruitment, included (i) the number of repeat episodes of cellulitis was added as an additional secondary outcome measure, as this had been omitted in error; (ii) exclusion criteria were amended to include conditions which prohibit the use of penicillin V, and having taken prophylaxis for the prevention of cellulitis within 6 months of the index episode. These changes were made in response to feedback received from regulatory authorities, the Trial Steering Group and the Data Monitoring Committee.

### Participants and setting

Recruitment took place in 20 hospitals between August 2006 and August 2008. Patients were identified either at presentation in secondary care (through emergency departments, medical admissions units, medical wards and outpatient clinics), or retrospectively via discharge coding or poster adverts.

Eligible participants had had a diagnosis of cellulitis of the leg within the last 12 weeks (index episode). Cellulitis was defined as a confirmed diagnosis of cellulitis by the recruiting dermatologist. If the patient was not seen by the recruiting clinician during the acute phase, then validation of the diagnosis was sought from medical case notes in combination with patient discussion. In this case, the following criteria were met: (i) local warmth and tenderness or acute pain; (ii) unilateral erythema, or asymmetrical erythema with the more severe side having a temporal relationship to the symptoms; and (iii) unilateral oedema. Any doubt over the certainty of the diagnosis was grounds for exclusion.

Patients were excluded from the trial if: (i) they had taken antibiotic prophylaxis for the prevention of cellulitis within 6 months of the index episode; (ii) it was longer than 12 weeks since the start of treatment for the index episode to the date of the baseline visit; (iii) they had known allergy to penicillin V; (iv) they had preceding leg ulceration, surgery or major penetrating trauma, because these cases were more likely to be caused by staphylococcal infection, which is not susceptible to penicillin V; (v) the recruiting clinician was unwilling to randomize the patient for medical reasons; (vi) they were aged < 16 years; (vii) they were unable to give informed consent; or (viii) they were already taking part in a research study.

### Interventions

Participants received either low-dose (250 mg twice daily) oral prophylactic penicillin V or placebo (250 mg twice daily) matched to the penicillin V tablets as far as possible by size and colour. Participants were asked to swallow the tablets whole to avoid tasting the penicillin V. A dosage of 250 mg twice daily was chosen in order to reflect current clinical practice among dermatologists (unpublished survey), and because the pharmacological properties of penicillin V suggest that twice-daily administration is preferable to once daily. Regardless of treatment allocation, participants stopped taking the trial medication if penicillin V was inappropriate (e.g. if they needed to take alternative antibiotics for an acute infection). Trial medication was re-started as soon as medically possible.

Participants who were identified for the trial while still receiving active treatment for their index episode were randomized once treatment of the index episode was complete.

Adherence to trial medication was assessed using self-reported tablet counts. These data were categorized as: 0, none taken; 1, hardly any taken (1–24%); 2, some taken (25–49%); 4, most taken (50–74%); 5, all taken (75–100%).

### Outcomes

Data collection was achieved through routine telephone follow-up calls from the coordinating centre (3-monthly during the treatment phase, and 6-monthly during the follow-up phase). Participants were asked to complete a study diary to record adverse events and health service resource use. If participants had a repeat episode of cellulitis they were asked to inform the trial staff immediately. Any repeat episodes not reported were picked up during the regular telephone interviews.

Health service resource use was captured using diaries and included inpatient admissions, outpatient visits, GP consultations, other prescribed drugs for cellulitis, and time away from normal daily activities.

The primary outcome measure for the trial was time to next episode of cellulitis. The next confirmed episode was defined as the next episode of cellulitis (in either leg) that had been reported by the participant, and confirmed by a medical practitioner. The episode was considered to have started on the first day of symptoms reported by the participant. Episodes that were reported by the participant and resulted in antibiotic treatment, but were not confirmed by a medical professional, were documented as self-reported cases and included in sensitivity analysis.

Secondary outcome measures included (i) the proportion of participants with repeat episodes of cellulitis at the end of the treatment phase, and at the end of the nonintervention follow-up phase; (ii) the number of repeat episodes of cellulitis; (iii) the proportion of participants with new oedema and/or ulceration at the end of the treatment phase, and at the end of the nonintervention follow-up phase; (iv) the number of nights in hospital for the treatment of repeat episodes of cellulitis; (v) the number of adverse drug reactions and/or adverse events of interest (death, nausea, diarrhoea, thrush, rash); (vi) cost-effectiveness; (vii) predictors of response model to explore the impact of known risk factors in predicting the efficacy of prophylaxis (to be conducted only if a significant treatment effect was found).

#### Changes to outcomes during the trial

Given the difficulties in achieving the target recruitment rate of 400 participants, some analyses that had been planned were subsequently dropped. These included (i) a planned subgroup analysis based on patients with and without recurrent cellulitis prior to entry into the trial; (ii) separate models for the predictors of response model based on ipsilateral and contralateral repeat episodes; and (iii) assessment of the impact of cellulitis on the quality of life of patients with cellulitis (which could be assessed only in those who were identified during the acute stage of the index episode). If possible, data from PATCH I and II trials will be combined to inform these analyses at a later date.

### Sample size

Sample size estimates assumed a 50% reduction in relapse rate relative to placebo based on a log-rank test for time-to-event data. This provided 80% power, at a two-sided significance level of 5%, with 20% loss to follow-up. A 50% reduction relative to placebo was determined as a minimum clinically useful gain, given the lengthy duration and possible inconvenience of long-term prophylaxis.

Previous studies suggest a range of possible recurrence rates for patients with cellulitis ranging from 30% to 50%,^1,19^ depending on the population being studied and the duration of follow-up. A conservative relapse rate for the placebo group of 25% was chosen, which resulted in a required sample size of 400 participants. Assuming that the relapse rate for those with recurrent cellulitis would be higher (35–50%), this also provided sufficient power to explore the planned subgroup analysis for patients with recurrent disease. However, following the decision to halt recruitment to PATCH II, this subgroup analysis was deemed to be exploratory only.

### Randomization and blinding

On confirmation that the index episode of cellulitis had resolved, participants were randomized by staff at the coordinating centre using a web-based randomization service provided by the Clinical Trials Unit (CTU) at the University of Nottingham. The computer-generated randomization list was produced prior to the start of the trial using randomly varying block sizes. Treatment allocations were concealed from all members of the trial team and were sent via e-mail to the pharmacy department at Queen’s Medical Centre, Nottingham, where the medications were dispensed using identical labelling and packaging, and posted to the participants’ homes.

Randomization was stratified by cellulitis status (first episode of cellulitis or more than one previous episode of cellulitis); by the presence or absence of pre-existing oedema; and by the presence of ulcer subsequent to the cellulitis.

The randomization list was held by the CTU. Participants and all members of the study team were blinded to treatment allocation throughout the trial, and analysis was performed prior to breaking of the randomization code. Although the treatments were packaged in an identical way, and the placebo tablets were of the same size and shape as penicillin V, the tablets were not identical due to difficulties in obtaining a matched placebo product (placebo tablets were unmarked and the penicillin V tablets were marked). Nevertheless, there was a low risk of unblinding as participants were recruited from a wide geographic area, with little or no contact with each other (thus minimizing the potential for unblinding by the direct comparison of active and placebo tablets). At the end of the study, participants were asked to record which treatment they thought they had received, in order to assess the success of blinding. Any treatment decisions requiring knowledge of the treatment allocation were referred to the data monitoring committee (DMC) or chief investigator, and systems were in place to allow for the breaking of the allocation code if required.

### Statistical analysis

Analysis of the primary outcome included all randomized participants with no exclusions (intention-to-treat). Time to recurrence was assessed using a Cox proportional hazards model and participants with limited follow-up data, including deaths, were included in the analysis but censored accordingly. Results presented are unadjusted (taken as the primary analysis), as well as adjusted for stratification factors.

Additional sensitivity analyses of the primary endpoint were planned: (i) including self-confirmed episodes of cellulitis; (ii) excluding randomized participants who did not start treatment or who reported a relapse within 4 weeks of randomization (such ‘relapses’ probably reflect incomplete treatment of the index episode rather than a true recurrence).

For the secondary outcomes: a test for nonproportional hazards was used to assess whether there was a significant change in treatment effect over time. The percentage difference in events between penicillin V and placebo for each treatment phase were calculated with 95% confidence intervals (CIs); the proportion of participants reporting multiple episodes were compared across treatment groups using a χ^2^ test for trend; the proportion of participants with new oedema and/or ulceration subsequent to enrolment in the trial were compared across treatment arms using a χ^2^ test and presented by treatment phase.

Although the planned subgroup analysis and predictors of response model were of limited value due to lack of power, exploratory analyses for the stratification factors (recurrent cellulitis/first episode cellulitis; presence of oedema and/or ulceration) are reported using appropriate tests for interaction.

Costs of care were estimated by applying published 2008/2009 national reference costs^20–22^ to the use of resources. CIs were estimated by bootstrapping with 1000 replications for each item.^23^ Two cost estimates are provided: NHS resources only, and a societal estimate which additionally values time lost from work or daily activities.

No interim analyses were planned or conducted. For all analyses 95% CIs are presented. Sensitivity analyses of the primary endpoint and analyses of secondary outcomes were considered to be supportive of the primary results, and so no adjustments for multiple testing have been made. All analyses were conducted in Stata, version 11.1 (StataCorp, College Station, TX, U.S.A.) statistical analysis software.

## Results

### Participants

**Fig 1:**
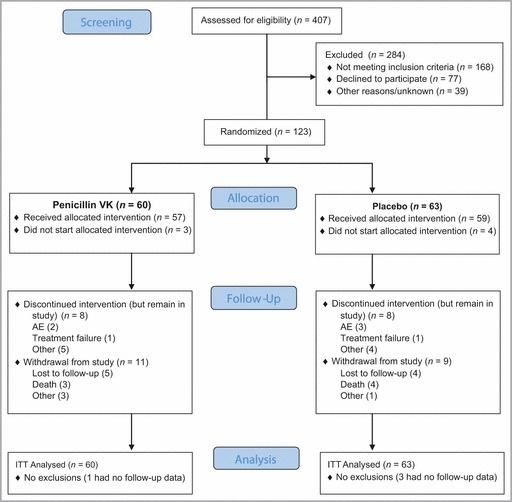
Participant flow diagram AE, adverse event; ITI, intent-to-treat.

Of the 407 participants screened, 123 were eligible and agreed to be randomized to the study (Fig. 1). This was lower than our target of 400 participants as the identification of suitable participants was much slower than anticipated, and recruitment was therefore stopped after 2 years due to funding limitations. The possible reasons for this failure to recruit have been reported elsewhere.^24^ In brief, many factors contributed to poor recruitment including: (i) changes in U.K. hospital policy, and the introduction of community-based care teams offering intravenous antibiotics meant that fewer patients with cellulitis were admitted to hospital than had been predicted; (ii) those who were admitted were seen by many different specialties, making it difficult for a network of dermatology clinicians to identify suitable participants; and (iii) without dedicated research nurses at the recruiting centres to support the principal investigators, it was extremely difficult to maintain the momentum of recruitment into the trial.

The majority of participants had no history of cellulitis prior to their index episode (79%) and almost half (49%) had no evidence of oedema or ulceration at baseline. These stratification factors and other baseline variables were broadly similar across the two treatment groups (Table 1).

**Table 1:** Baseline characteristics, *n* (%) unless otherwise specified

	Penicillin V, *n *=* *60	Placebo, *n *=* *63
*Stratification factors*		
Previous cellulitis		
Yes	12 (20)	14 (22)
No	48 (80)	49 (78)
Condition		
No evidence of oedema or ulcer	30 (50)	30 (48)
Presence of pre-existing oedema	27 (45)	29 (46)
Ulcer subsequent to cellulitis	2 (3)	2 (3)
Both pre-existing oedema and ulcer	1 (2)	2 (3)
*Demography*		
Age		
Mean (SD)	56·7 (14·0)	59·5 (14·6)
Median, IQR, range	58, 48–66, 18–81	60, 49–71, 23–84
Sex		
Male	22 (37)	20 (32)
Female	38 (63)	43 (68)
*Diagnosis of cellulitis*		
Confirmed as cellulitis by dermatologist	60 (100)	63 (100)
Local warmth/tenderness/acute pain		
Yes	60 (100)	63 (100)
No	0 (0)	0 (0)
Erythema at the affected site		
Yes	60 (100)	62 (98)
No	0 (0)	1 (2)
Oedema at the affected site		
Yes	60 (100)	63 (100)
No	0 (0)	0 (0)
*Risk factors for cellulitis*		
Body mass index		
Mean (SD)	34·0 (9·8)	31·0 (6·8)
Median (IQR)	32·3 (27·6–38·4)	29·8 (26·4–34·1)
Asymmetrical chronic oedema/lymphoedema		
Yes	19 (32)	27 (43)
No	41 (68)	36 (57)
Symmetrical chronic oedema/lymphoedema		
Yes	10 (17)	5 (8)
No	47 (78)	55 (87)
Unknown	3 (5)	3 (5)
Venous insufficiency		
Yes	12 (20)	15 (24)
No	46 (77)	47 (75)
Unknown	2 (3)	1 (2)
Leg ulcer subsequent to cellulitis		
Yes	4 (7)	5 (8)
No	56 (93)	58 (92)
Tinea pedis/toeweb maceration		
Yes	24^a^ (40)	19^a^ (30)
No	36 (60)	43 (68)
Unknown	0 (0)	1 (2)
Preceding surgery to the leg > 2 weeks ago		
Yes	11 (18)	4 (6)
No	44 (73)	51 (81)
Unknown	5 (8)	8 (13)
Blunt injury		
Yes	5 (8)	7 (11)
No	54 (90)	56 (89)
Unknown	1 (2)	0 (0)
Intravenous drug abuse		
Yes	0 (0)	0 (0)
No	60 (100)	63 (100)
Diabetes		
Yes	14 (23)	12 (19)
No	45 (75)	51 (81)
Unknown	1 (2)	0 (0)
Onychomycosis		
Definitely	2 (3)	2 (3)
Probably	11 (18)	12 (19)
No	46 (77)	49 (78)
Unknown	1 (2)	0 (0)
Treatment of index episode		
Inpatient admission	48 (80)	45 (71)
Mean days in hospital (including all participants)	5·3 (5·4)	8·7 (10·3)

IQR, interquartile range. ^a^Number with treatment prescribed for tinea pedis at baseline [penicillin V = 19/24 (79%); placebo = 17/19 (89%)].

Of the 123 participants randomized, 20 (16%) participants (11 penicillin V and nine placebo) did not reach the end of the study (Fig. 1). Participants in both groups had a similar study time experience, and approximately 80% had at least 2 years of follow-up.

### Treatment adherence

From self-reported tablet counts, 97 (79%) patients fully adhered to treatment, defined as at least 75% of tablets taken. This was similar across treatment groups (48 penicillin V and 49 placebo). Seven participants did not start treatment (three penicillin V and four placebo).

At the end of the trial, 101 participants provided a guess as to what treatment they were on. Of these, 42% were unsure, 37% guessed penicillin V (of whom 73% were correct) and 22 guessed placebo (of whom 77% were correct). For those who guessed correctly, this was based on whether or not they had experienced a recurrence of cellulitis. Only 13 correct guesses related to recognizing the smell, taste or look of penicillin V (or lack of for placebo).

### Primary outcome: time to first confirmed recurrence of cellulitis

**Fig 2:**
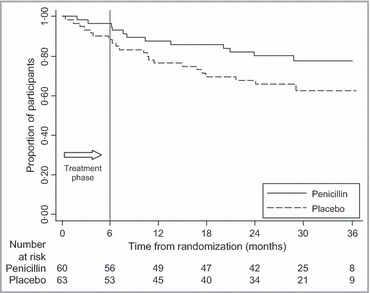
Time to first recurrence of cellulitis.

Results from the Cox proportional hazards model analysis resulted in an estimated 47% reduction in the risk of confirmed recurrence of cellulitis, HR = 0·53, 95% CI (0·26–1·07); *P* = 0·08 (Fig. 2). In the penicillin V group, 12 (20%) patients experienced a recurrence compared with 21 (33%) in the placebo group (Table 2). This equates to a number needed to treat (NNT) of eight participants in order to prevent one repeat episode of cellulitis [95% CI NNT(harm) 48 to ∞ to NNT(benefit)3].

**Table 2:** Primary analysis: time to first repeat episode of cellulites

	*n* events/*n* patients randomized	HR (95% CI)	*P*-value	Adjusted HR^a^ (95% CI)	*P*-value
Primary analysis (ITT): repeat episodes confirmed by medical practitioner					
Penicillin V	12/60	0·53 (0·26–1·07)	0·08	0·51 (0·25–1·05)	0·07
Placebo	21/63	1			
Total	33/123				
Sensitivity analyses					
Including unconfirmed episodes (self-reported)					
Penicillin V	14/60	0·60 (0·31–1·17)	0·14	0·58 (0·30–1·13)	0·11
Placebo	22/63	1		1	
Total	36/123				
Excluding participants who never started treatment, or had a relapse within 4 weeks of index episode					
Penicillin V	12/57	0·57 (0·28–1·16)	0·12	0·55 (0·27–1·12)	0·10
Placebo	20/58	1			
Total	32/115				

CI, confidence interval; HR, hazard ratio. ^a^Adjusted for stratification factors (pre-existing oedema/ulceration).

On adjustment for stratification factors, the result was similar: HR = 0·51, 95% CI (0·25–1·05), *P* = 0·07, as were the results of the sensitivity analyses (Table 2).

### Secondary outcomes

The protective effects of penicillin V in preventing recurrence of cellulitis did not vary between the treatment phase and nontreatment follow-up phase of the trial (nonproportional hazards, *P* = 0·7). The difference in relapse rates during the treatment phase was −8%, 95% CI (−17% to 1%): 2/60 (penicillin V) and 7/63 (placebo). During the post-treatment phase, the between-group difference was −9%, 95% CI (−24% to 7%): 10/56 (penicillin V) and 14/53 (placebo).

Of the 33 participants with at least one confirmed episode of cellulitis, three had two repeat episodes, three had three, two had four, and one had seven repeats. There was no significant difference in the total number of repeat episodes between the treatment groups (*P*_trend_ = 0·10).

Sixty participants had no oedema and/or ulceration at baseline. Of these, 35 (58%) developed oedema and/or ulceration during the trial, although only nine experienced a repeat episode. There were no significant between-group differences in the presence of oedema or ulceration either during the treatment phase (penicillin V 37% vs. placebo 21%; *P* = 0·31), or overall (penicillin V 67% vs. placebo 53%; *P* = 0·40).

During the trial, 36 participants experienced at least one of the prespecified adverse events of interest. None of the seven deaths were considered to be treatment or trial related. The total number of adverse events of special interest was similar for both treatment groups: nausea (13 penicillin V and 11 placebo); diarrhoea (14 penicillin V and 18 placebo); thrush (three penicillin V and three placebo); rash (four penicillin V and three placebo). No suspected, unexpected, serious adverse reactions (SUSARS) were reported during the trial.

### Exploratory analyses

No difference was found in the effect of penicillin V on the primary endpoint between those with a history of previous cellulitis [HR = 0·38, 95% CI (0·11–1·27)] and no previous history [HR = 0·60, 95% CI (0·25–1·44), *P*_interaction_ = 0·58]. Similarly, no difference in the effect on the primary endpoint was found between those with oedema and/or ulceration at baseline [HR = 0·48, 95% CI (0·16–1·43)] and those without [HR = 0·58, 95% CI (0·23–1·50), *P*_interaction_ = 0·79].

### Resource use and cost

There were no significant differences between the groups in any resource items or costs (Table 3). Overall there was a nonstatistically significant reduction in NHS costs favouring penicillin V: (penicillin V – placebo) −£93, 95% CI (−£372 to £128), *P* = 0·45, as well as in broader societal costs: −£215, 95% CI (−£1155 to £707), *P* = 0·67 (Table 3). A full cost-effectiveness analysis for PATCH I and II will be published separately.

**Table 3:** Patient level analysis of resource use and cost

	Penicillin V	Placebo	Δ	95% CI	*P-*value
Net resource use					
GP visits	2·93	3·56	−0·6	−6·7 to 3·3	
Community visits	0·42	2·48	−2·1	−6·6 to 0·8	
Inpatient stays	0·02	0·05	−0·03	−0·12 to 0·03	
Outpatient visits	0·17	0·14	0·02	−0·28 to 0·29	
Missed employment (days)	5·32	6·56	−1·2	−10·5 to 7·7	
Net cost (£, 2009)					
Trial antibiotic	18	–	18		
Nontrial prescribed drugs	8·10	5·30	3	−5 to 13	0·57
Total, NHS	192·40	186·70	−93	−372 to 128	0·45
Total, societal	713·40	928·10	−215	−1155 to 707	0·67

CI, confidence interval. Δ, mean difference: penicillin V – placebo; up to 3-year data.

The number of hospital admissions for the treatment of recurrent episodes was low in both groups. Of the 36 repeat episodes recorded during the trial, only three resulted in a hospital admission (two in penicillin V; one in placebo).

## Discussion

This trial suggests that it is highly probable that a substantial reduction in the number of repeat episodes of cellulitis of the leg could be achieved by giving patients prophylactic antibiotics for a period of 6 months after treatment of the acute episode. The result was of borderline statistical significance (meaning that there is an 8% chance that the observed benefits could have occurred by chance), but has to be interpreted in the context of a virtual absence of similar data elsewhere, the large potential magnitude of effect, and the consistency of possible benefit for a range of outcomes.^25^ The study indicates that a possible treatment effect deserves further investigation, especially as the intervention is low cost, safe and well tolerated by patients.

Although the PATCH II trial suggested a large treatment effect (a 47% reduction in the risk of a repeat episode), prophylactic penicillin did not prevent all subsequent cases of cellulitis of the leg. This would suggest that other factors are also important in determining whether or not a patient will experience further episodes. This is consistent with other studies, which report that penicillin treatment may not achieve microbial clearance^26^ and that even when prophylaxis is ongoing, some patients continue to experience further attacks.^27^

The findings are particularly important as they challenge two commonly held beliefs about the management of cellulitis: (i) that prophylactic penicillin V is warranted only in people with recurrent cellulitis and/or those who have known risk factors for repeat attacks, such as lymphoedema; and (ii) that prophylactic antibiotics are required long-term (or indefinitely) for benefits to be sustained. If the findings of the PATCH II trial are replicated in other studies then it is possible that all patients could be routinely offered a 6-month course of low-dose penicillin V after an attack of cellulitis of the leg. The rationale for such a treatment option is sound. Previous researchers have demonstrated that lymph drainage is compromised following an attack of cellulitis.^28,29^ It is therefore possible that a typical 7–10-day course of antibiotics during the acute phase of the infection may not be sufficient to achieve complete microbial clearance from the lymph system. The traditional model of giving antibiotic prophylaxis only to patients with recurrent cellulitis, or to those who already have chronic lymphoedema may in fact be too late to prevent the permanent impairment to lymph drainage in the leg that ensues following repeated episodes of cellulitis.

Penicillin has been used as long-term medication for many years in other conditions such as rheumatic fever, and group A streptococcus has remained susceptible to penicillin for over 60 years without signs of developing resistance.^30^ It therefore represents a very cheap intervention (£18 per 6-month course) that has potential for substantial health savings. Assuming a NNT of 8, this equates to a treatment cost of £144 per episode of cellulitis prevented.

It is disappointing that the trial failed to achieve its recruitment target of 400 participants and thus failed to provide sufficient evidence on which to base firm conclusions. Nevertheless, the trial does suggest a potentially large protective effect that was consistent throughout the follow-up period, and robust in sensitivity analysis.

Future cellulitis trials can benefit from the experiences of the PATCH II trial in designing trials that might recruit patients with cellulitis more successfully. Indeed, the PATCH I trial, which is due to report in early 2012, introduced modifications to the trial design and conduct as a result of our experiences with PATCH II. This trial successfully recruited 274 participants (105% of the original sample size requirement). Changes to the protocol included (i) amending the eligibility criteria so that patients who had had an episode of cellulitis within the last 6 months (rather than 3 months) were able to take part; (ii) amending the case report forms so that recruiting clinicians were able to concentrate on essential medical information, with more routine clinical trial data being collected during telephone contact between the coordinating centre and the participants prior to randomization; (iii) advertising for participants in local media (radio, websites and newspaper advertorials). Setting up dermatology department based cellulitis services treating lower limb cellulitis with once-daily intravenous antibiotics (usually at home or as outpatients) may improve diagnostic accuracy and provide a source of recruitment for future studies.^3^

Notwithstanding under-recruitment, this was a well-conducted trial, with blinded outcome assessment and rigorous allocation concealment. The follow-up period of up to 3 years was sufficient to capture the treatment effect and loss to follow-up was low. Self-reported adherence and safety monitoring would suggest that the treatment was well tolerated and that patients adhered to the treatment schedule successfully.

The PATCH II trial was designed as a pragmatic trial that aimed to reflect current practice as far as possible. Eligibility criteria were broad and contact with health professionals was kept to a minimum as would be the case in normal practice. Recruitment into the trial was conducted in 20 hospitals throughout the U.K. and southern Ireland and should therefore be representative of the type of patients seen in secondary care. However, it is likely that recruited patients had more severe disease than those typically seen in a primary care setting.

This is the first trial to have explored the use of medium-term (6-months) antibiotic prophylaxis in patients who have had cellulitis of the leg. Current clinical guidelines are based on very limited trial evidence and so this trial represents a potentially valuable addition to clinical knowledge. The results of the PATCH I trial (available early 2012, and based on 274 participants) will hopefully shed further light on the use of prophylactic antibiotics for the prevention of cellulitis in patients with recurrent disease.

**What’s already known about this topic?**

- Cellulitis/erysipelas is very common, and is associated with high patient morbidity and health service costs.
- Three small trials suggest a possible benefit of antibiotic prophylaxis in patients with recurrent disease.
- Current national guidelines are based on limited trial data and professional consensus.

**What does this study add?**

- The study suggests that taking oral penicillin V (250 mg twice daily) for 6 months after an episode of cellulitis of the leg may reduce further attacks.
- These findings warrant further study, and consideration for provisional inclusion in clinical guidelines given the absence of better data on this important topic.
